# Challenges to Achieving Surgical Equity in Slums

**DOI:** 10.3389/ijph.2024.1608098

**Published:** 2025-01-13

**Authors:** Rahul M. Jindal, Sushila Tiwari

**Affiliations:** ^1^ Uniformed Services University of the Health Sciences, Bethesda, MD, United States; ^2^ Indian Institute of Public Health Gandhinagar, Gandhinagar, India; ^3^ Office of Human Rights, Rockville, MD, United States; ^4^ Strayer University, Herndon, VA, United States

**Keywords:** unmet surgical needs, slum surgical health, slum health, surgical equity, India

## Abstract

There is a critical lack of surgical data on individuals who live in urban slums, which hampers the allocation of healthcare resources and the provision of preventative measures. The complex interplay of factors affecting surgical care in slums, such as trust deficits, mental health concerns, and socioeconomic barriers, necessitates a distinct academic approach. We propose that researchers should consider “slum surgical health” as an area of study separate from urban health or slum health. From this perspective, we make a case for defining “slum surgical health” while presenting evidence from multiple countries that shows the unique challenges of providing surgical care in slum settings. We discuss a successful model that has deployed community health worker programs as intermediaries between slum dwellers and healthcare providers. This model, which achieved a 60% conversion rate from unmet to met surgical needs, demonstrates the potential of culturally sensitive, community-based approaches to address surgical inequities in urban slums.

## Introduction

The United Nations projects the world’s urban population to grow by 2 billion before 2030; nearly one-third (32%) of the world’s population lives in slums, with more than 90% of the growth occurring in Low- and Middle-income countries (LMICs) [1]. The location of slums is generally in polluted environments which makes them uniquely susceptible to major calamities such as the disaster from a pesticide factory in Bhopal, India, which killed more than 20,000 slum residents.

Some view the word “slum” as pejorative, and it frequently has social and emotional connotations and is used derogatorily. However, international agencies, governments, civil society, and scholars continue to use the word. Street and pavement dwellers are not considered part of slums in official censuses and rehabilitation projects.

The central theme of our manuscript is to highlight the current surgical inequity in urban slums in LMICs and to encourage research on this neglected population. We suggest several ways to achieve surgical health equity in LMIC slums one of which is to use specifically trained community health workers as liaisons between the community and healthcare facilities. Developing programs to reduce surgical inequity must be accompanied by poverty alleviation, legalization of slums, and provision of adequate sanitation and primary healthcare under a universal healthcare system. There is also an urgency to have a baseline assessment of Surgical Care Indicators as suggested by The Lancet Commission on Global Surgery and a National Surgical, Obstetric, and Anesthesia Plan (NSOAP) [2] to provide surgical care to 1.4 billion individuals in India, including those who reside in slums. Data accrued from slum surgical health could be used by policymakers crafting health budgets, healthcare administrators managing resource allocation, urban planners designing accessible facilities, and public health researchers studying the effectiveness of interventions.

## Definition of Slum

The word “slum” was first used in the early part of the 19th century in London to describe a room of low repute or low, unfrequented parts of the town. The United Nations has coined a specific definition of a slum as one or a group of individuals living under the same roof in an urban area and lacking one or more of the following five amenities; durable housing; sufficient living space; sufficient access to water; access to adequate sanitation and secure tenure.

The Government of India has invested considerable resources in identifying and classifying the approximately 400 million individuals who reside in slums as part of its policy to alleviate poverty and health inequities, with moderate success. In India, slum settlements have to be legalized to enable them to have potable water and sanitation. These projects fall administratively under the *Rajiv Awas Yojana*, which envisions a “slum-free” India [3].

Slum is often called *chawl* in Western India, *bustee* or *jhuggi* or *zopadpatti* in other parts of India or favela in Portuguese and in tugurio, chabola, or barriada in Spanish.

## Urban Health and Slum Health

The terms urban health and slum health are often used interchangeably in the literature. Studies have shown that there is a knowledge gap about the urban poor and their health status [4]. The study of slums is technically difficult because they expand rapidly. Most concerning is that 90% of this slum growth is concentrated in LMICs. This pattern is evident across multiple regions, as demonstrated by Hyderabad, India, where there was a 70% increase in the slum area between 2003 and 2010. Similar expansions are occurring across all LMICs, with striking examples in several cities such as Nairobi, Kenya, where more than 60% of the urban population (approximately 2.5 million individuals) lives in informal settlements that occupy just 6% of the city’s land area. In Ghana’s capital Accra, informal settlements have expanded rapidly, with approximately 58% of the urban population (about 2.2 million individuals) living in slum conditions as of 2020. Malaysia presents a different pattern of urban poverty, with an estimated 25% of Kuala Lumpur’s population (approximately 1.7 million individuals) living in informal settlements or low-cost housing. Bangladesh’s capital Dhaka shows perhaps the most dramatic urban transformation, with more than 40% of its 21 million inhabitants living in slums.

Inadequate sanitation systems and poor drainage infrastructure create conditions ripe for disease transmission. Environmental health risks abound, including contaminated water sources, indoor air pollution from cooking fires, exposure to industrial waste, and vulnerability to flooding. These infrastructure deficiencies directly impact surgical outcomes and recovery conditions. Indoor air pollution (IAP) is frequently seen in slums due to smoke released from cooking on open fires without adequate ventilation. This has been recognized by the World Health Organization as a major environmental health risk, responsible for approximately 4 million deaths in 2012. Exposure to IAP has been linked to acute pneumonia, chronic obstructive pulmonary disease, lung cancer, cardiovascular disease, and others. Poor water quality is a leading cause of morbidity and mortality in slum dwellers causing life-threatening diseases such as cholera and hepatitis. Lack of access to water also restricts sources for infant feeding or cooking, bathing, and personal hygiene.

## Effect of Climate Change on Slum Health

Urban slums are uniquely susceptible to climate change due to their location on flood plains or toxic waste sites [5]. Contamination of groundwater, poor sanitation, and overcrowding lead to infectious and non-communicable diseases, violence and injuries, and mental health issues. Slum dwellers live in crowded areas which exacerbates the transmission of tuberculosis and domestic injuries. The surgical burden of neglected tropical diseases (NTDs) is predicted to rise alongside average temperatures and drought. These include trachoma and lymphatic filariasis which have surgical manifestations and predominantly affect individuals in LMICs where the effects of climate change are most severe. Climate change will certainly lead to sociopolitical destabilization while a lack of investment in healthcare infrastructure will jeopardize the fight against NTDs and threaten the achievement of the 2030 goals.

## An Indian Model to Reduce the Burden of Surgical Diseases in Slums

The project in India was initiated in 2018, the same year of India’s Universal Health Coverage (UHC), dubbed “Modicare” by the media. This is touted to be the world’s biggest government-funded health insurance program covering more than 100 million of India’s poorest families, with 40% of the population receiving free health coverage for secondary and tertiary hospitalization [6].

The estimated unmet surgical needs are high in LMICs; however, few studies have been conducted. Our team in collaboration with a local NGO https://saath.org/ and the Indian Institute of Public Health Gandhinagar https://iiphg.edu.in/ implemented a stepwise series of studies to first identify the burden of surgical disease in a slum; the reasons why slum dwellers do not seek treatment under India’s existing UHC; compare the burden of surgical disease in a rural Tribal area vs. a slum in Ahmedabad (a Metropolitan city of approximately 8.3 million individuals of which approximately 20% live in legally designated slums); create a curriculum for specialized community health workers (CHWs), and conduct a pilot to study their effectiveness.

1. Survey of surgical unmet needs in a slum of a metropolitan city in India: We first established a baseline by investigating the burden of surgical conditions, level of unmet need, and reasons for non-utilization of surgical services in a slum of Ahmedabad, a metropolitan city in western India. A community-based cross-sectional study was carried out that showed that out of 10,330 individuals in 2066 households, 3.46% (n = 357) patients needed surgery; 1.46% (n = 150) did not seek surgery and were categorized as “unmet need.” This study showed that the majority of individuals with surgical needs had abdominal- or extremities-related problems followed by the need for eye surgery; back, chest, and breast. The most striking finding of this study was that all participants had obtained a consultation, however, approximately half of the patients recommended for surgery did not receive the surgical care available under Modicare mostly because of perceived financial reasons and trust deficits [7].
2. Trust deficit in surgical systems in an urban slum: Following the initial study of unmet surgical needs in the urban slum, we carried out a mixed-method study to understand why patients did not seek surgical care despite the widespread availability of UHC. This was a follow-up study to understand the reasons for not seeking surgery. We administered standardized instruments and conducted in-depth interviews and focus groups. Responses showed that although patients thought that physicians were well qualified and hospitals were of good quality, there was a significant trust deficit in actualizing prescribed surgical care [8]. This stems from their experiences of displacement, previous negative interactions with healthcare professionals, fears of discrimination, and limited understanding of medical procedures. Trust deficits represent significant barriers to accessing available surgical care.
3. Unmet surgical needs and trust deficits in marginalized communities: There is a perception that unmet surgical needs are higher in rural versus urban areas in LMICs. We, therefore, compared unmet surgical needs and trust in physicians and perceptions of health system quality in a rural Tribal area and an urban slum in India. We surveyed 7914 individuals in 2066 households in urban slums and 5180 individuals in 1036 households in rural Tribal areas. We used the Surgeons Overseas Assessment of Surgical (SOSAS) to identify met and unmet surgical needs and used two validated instruments to study perceptions of healthcare. We found that unmet surgical needs in Tribal areas were less than 5% versus 39% in the urban slums. Surgical unmet needs were significantly lower in Tribal versus urban areas, possibly due to the high priority given by the Indian government to alleviating poverty, and social deprivation and the vigorous participation of NGOs [8].
4. Development of a short curriculum for the Surgical Accredited Trained Healthcare Initiative (SATHI): As a result of the survey in the slums and rural areas of the Western state of Gujarat, our team concluded that there was a need for specially trained CHWs to act as intermediaries to educate and liaise between the slum dweller who needs surgical care and the surgical provider. We designed a short, one-week curriculum that would enable a CHW to identify common surgical problems based on symptomatology [9].
5. A pilot study of SATHI to reduce unmet surgical needs in an urban slum: Our work with CHWs - SATHI as an intermediary channel, links slum dwellers to service providers [10]. The concept of SATHI derives its profound significance from its Sanskrit etymology, where “Sathi” (साथी) means “companion” or “friend.” This meaning finds deep resonance in various religious and cultural traditions, each of which emphasizes the sacred nature of friendship.

In Hindu scripture, particularly the Bhagavad Gita, the divine friendship between Krishna and Arjuna serves as the ultimate example of guidance and support. Krishna, acting as both friend (सखा/sakha) and mentor, guides Arjuna through his moral crisis on the battlefield. In the Bible, the concept of friendship is exemplified through multiple stories. The parable of the Good Samaritan (Luke 10:25-37) demonstrates how true companionship transcends social boundaries, particularly in times of medical need. The friendship between David and Jonathan (1 Samuel 18:1-3) illustrates unconditional support and loyalty, with scripture stating their souls were “knit together.” The Qur’an emphasizes the importance of friendship and mutual support, particularly in Surah Al-Hujurat (49:10), which declares “The believers are but brothers.”

These religious perspectives on friendship align perfectly with SATHI’s mission to provide trusted healthcare companions who offer guidance on complex medical decisions; provide emotional support during vulnerable times; help navigate healthcare systems; build bridges between communities and medical providers and ensure continuity of care through personal connection.

CHWs are an all-female workforce with only an 8th-grade education who themselves reside in the slums. SATHIs help patients attain the benefits of healthcare schemes, and offer counseling by building trust and reducing fear of surgical procedures. SATHIs also provide emergency first aid for minor injuries commonly seen in daily wage laborers, and measure BP and blood sugar. As a first step, we trained six SATHIs by administering the basic unmet surgical needs curriculum. We then carried out a pilot study in the slums of Ahmedabad, a metropolitan city in India, to evaluate their effectiveness in reducing the burden of unmet surgical needs. The study comprised 12, 730 individuals residing in 3000 households over 6 months. Our study showed that there were 126 subjects (0.98%) with met needs and 167 (1.31%) with unmet surgical needs. Most importantly, SATHIs were able to move 99 patients (59.28%) from unmet to met needs, as they received surgery/treatment. However, 68 patients (40.71%) were still in the unmet category even after counseling. This moderate success was achieved by building trust, facilitating access to healthcare, and ensuring post-operative adherence [11]. In consultation with the community, we scaled up the project to 350,000 slum dwellers in 70,000 homes.

## Suggested Strategies to Reduce the Burden of Surgical Disease in Slums

The determinants of slum health are multi-factorial arising from a combination of poverty, climate change, and marginalization from the mainstream society.

1. Gather robust data on the burden of surgical disease: It is difficult to obtain accurate health statistics in slums due to mobile populations, trust deficits, poor literacy, crime, and alcoholism – likely the tip of the iceberg. The majority of slum disease burden and mortality data are based on outpatient clinic, hospital admissions, or national mortality registry data which underestimate the underlying medical conditions.
2. The need for a new analytical framework to understand surgical health in slums: The Lancet Commission on Global Surgery (LCoGS) suggested tracking six indicators for universal surgical, obstetric, trauma, and anesthesia care by 2030, namely preparedness for surgical, anesthesia, and obstetric care (timely access to care and availability of SAO workforce), delivery of surgical, anesthesia, and obstetric care (surgical operative volumes and perioperative mortality rates), and effect of surgical, anesthesia, and obstetric care (financial risk protection or lack thereof against catastrophic and impoverishing health expenditures among surgery needing populations). Zadey et al. [2] reviewed the literature to investigate the status of LCoGS indicators in India and found that there was a lack of data on access to timely essential surgery, risk of impoverishment and catastrophic health expenditures due to surgery and the surgical specialist workforce. Furthermore, estimates were heterogeneous across urban and rural areas. They concluded that India needs a subnational mapping of indicators disaggregated for rural, tribal, urban, public and private (non- and for-profit) health sectors. Synthesizing disaggregated indicators in urban slums would be crucial to understanding India’s needs for equitable surgical care planning.
3. Target modifiable conditions of slum life: Slum-specific information will lead to an improved share of national and regional budgets so that low-cost solutions such as closing open sewers to limit diarrheal disease, litter-free footpaths with adequate street lighting to deter violence, constructing protective barriers to prevent traumatic injuries, or reinforcing infrastructure to protect slum dwellers from environmental hazards can be trialed. Community-based interventions should include participatory maintenance initiatives and disaster preparedness protocols that when combined with evidence-based infrastructure improvements, demonstrate the potential for sustainable health outcomes in these vulnerable populations. This data-driven approach enables policymakers to allocate resources effectively while addressing both immediate health concerns and long-term community resilience.
4. Implement simple solutions first: Health professionals can assume leadership roles by organizing neighborhood associations and civil societies in the era of social media and the widespread availability of the internet and smartphones. Simple solutions such as hand-washing with soap can reduce the risk of diarrheal diseases by up to 47% and could save one million lives in the slums. To prevent burn injuries in slums, several strategies can be implemented, such as installing protective barriers around communal cooking zones, implementing secure storage systems for flammable cooking fuels, and establishing supervised childcare facilities within the community. Simple infrastructure modifications like elevated cooking platforms and properly secured stoves can significantly reduce the risk of accidental burns, particularly among unsupervised children in densely populated areas. Community-based interventions should include training local volunteers in fire safety monitoring and basic first aid response, while educational programs in neighborhood schools can help cultivate a culture of burn prevention awareness. These measures, combined with rapid evacuation protocols to nearby burn centers, form a comprehensive approach to reducing burn-related morbidity in space-constrained slum environments.
5. Devise specific therapy for the emotional vulnerability of displaced populations: The emotional landscape of slum dwellers is marked by perpetual uncertainty. These displaced populations face constant instability in their housing situation, leading to a fundamental distrust of institutions. The looming threat of eviction prevents long-term healthcare planning, while the absence of established social support systems in their new urban environment further compounds their vulnerability.
6. Assessment of mental health challenges in surgical care: Mental health considerations play a crucial role in decisions about surgical care among slum dwellers. The psychological burden is amplified by practical worries about post-surgical support and recovery. The findings consistently show that psychological barriers, combined with economic constraints, significantly impact surgical care decisions.
7. Introduce the term “*slum surgical health*” as an area of study that is distinct from urban health or slum health. The academic discipline of Global Surgery is expansive and seeks to provide improved and equitable surgical care to the world’s population, based on the central pillars of need, access, and quality. However, this is an emerging terminology that generally conveys projects carried out in rural or austere settings ravaged by war or natural calamities. There is very little discussion on the burden of surgical disease in slums in the literature. Public-private partnerships may also be an option in which government funding is minimal. Surgical trainees could potentially work in the urban slums in LMICs to compensate for the decline in volume, variety, and open surgical cases under supervision and stringent ethical guidelines [12].
8. Need for a consensus conference on surgical health in slums: The *Karad Consensus Statement* at the National Surgical Forum made specific recommendations to scale up surgical care in rural areas of India which comprise over 65% of the Indian population [13]. There is now an urgent need for a consensus statement on slum surgical care along the lines of the Karad statement.

### Conclusions

A multi-pronged approach to reducing the burden of surgical disease in slums will require the combined efforts of the Indian government, civil society, and NGOs. This approach must address both structural and psychosocial barriers to surgical care, including:

1. Legalization of slums and provision of basic amenities: Legal recognition enables slum dwellers to access government services and healthcare benefits without fear of eviction. Basic amenities like water, electricity, and proper housing create conditions conducive to better surgical outcomes and recovery.
2. Implementation of poverty alleviation programs: Economic stability allows families to consider surgical treatment without fear of catastrophic financial consequences. Poverty alleviation programs may include job training, microfinance initiatives, and direct financial assistance for healthcare needs.
3. Development of adequate sanitation infrastructure: Proper sanitation reduces the risk of post-surgical infections and improves overall community health outcomes. Infrastructure development includes sewage systems, waste management, and access points to clean water.
4. Establishment of comprehensive primary healthcare systems: Primary healthcare centers serve as the first point of surgical screening and referral in slum communities. These centers can provide basic surgical procedures and coordinate with larger hospitals for complex cases.
5. Addressing trust deficits through community engagement: Building trust requires a consistent community presence and culturally sensitive healthcare delivery approaches. Community health workers who come from the same communities can bridge the gap between medical institutions and slum residents.
6. Supporting mental health needs related to surgical care: Mental health support helps patients overcome fears and anxieties about surgical procedures and recovery. Counseling services and support groups can address concerns about job loss, family responsibilities, and post-surgical rehabilitation.
7. Creating sustainable financial support mechanisms: Financial mechanisms should include both government insurance schemes and community-based funding solutions. Sustainable support systems must consider both immediate surgical costs and long-term recovery expenses.

The SATHI model of specialized CHWs, with its structured curriculum, adequate supervision, and compensation, has proven effective in managing surgical disease in slum settings [14]. This model, which combines technical training with cultural sensitivity and trust building, could serve as a template for similar initiatives in other LMIC settings.

## Abbreviations

LMIC, Low and Middle-Income Countries; IAP, Indoor air pollution; NTDs, neglected tropical diseases; DALYs, disability-adjusted life years; SATHI, Surgical Accredited and Trained Healthcare Initiative; CHW, Community health workers; NSOAP, National Surgical, Obstetric and Anesthesia Plan; UHC, Universal Health Coverage, LCoGS, The Lancet Commission on Global Surgery.
